# Draft Genome Sequence of Ureolytic Environmental Isolate *Staphylococcus* sp. NA309

**DOI:** 10.1128/genomeA.01066-16

**Published:** 2016-10-06

**Authors:** Jonathan R. Gaiero, Tom Hsiang, Rob W. Nicol, Marc Habash

**Affiliations:** School of Environmental Sciences, University of Guelph, Guelph, Ontario, Canada

## Abstract

We report the 2.7 Mb draft genome sequence of *Staphylococcus* sp. NA309 isolated from poultry litter. The isolate was a dominant member of the cultivable aerobic bacteria identified to have ureolytic activity, responsible for ammonia generation in poultry litter residue.

## GENOME ANNOUNCEMENT

*Staphylococcus* sp. NA309 was isolated in 2013 from a survey of cultivable aerobic bacteria present in poultry litter in southern Ontario, Canada (1). Isolate NA309 was identified as ureolytic based on a color change on Christensen’s urea agar indicator medium. PCR amplification and sequencing was performed on the alpha subunit (*ureC*) of the urease CDS and was identified as closely matching the PLUP group of urease sequences (2). This group, previously identified only by culture-independent sequencing, was found to be a dominant group in several poultry litters tested in the United States (2). *Staphylococcus* spp. are common in poultry litter (3), and known to be ureolytic (*Staphylococcus equorum* subsp. *linens*, *S. succinus* subsp. *succinus*) (4, 5). Based on identification by 16S rRNA gene sequencing, *Staphylococcus* sp. NA309 was determined to be the dominant member of the cultivable ureolytic bacteria, comprising 25% of the community.

Genomic DNA from NA309 was isolated from a pure culture grown in nutrient broth at 30°C. DNA was extracted using the DNeasy blood and tissue kit (Qiagen, Germantown, MD) according to the manufacturer’s recommended protocol for Gram-positive bacteria. Two micrograms of DNA were sent for sequencing using an Illumina HiSeq 2000 platform and 100-bp paired-end reads at the Genome Quebec facility in Montreal, Quebec, Canada. A total of 22,200,944 reads were generated, with a genome sequence coverage of 1,531×. Genome assembly was performed using ABYSS (version 1.5.2), generating 54 contigs. The draft genome comprised 24 contigs that were greater than 200 bp in length, was 2,730,867 bp in size, with an average G+C% content of 33.1%. The NCBI prokaryotic genome pipeline (GeneMarkS+ version 2.1) was used to predict a total of 2,491 protein-encoding genes, 16 rRNA genes, 38 tRNA genes, and 106 pseudogenes.

The scaffold assembly was also uploaded to the Rapid Annotation using Subsystem Technology (RAST) (6) (http://rast.nmpdr.org/rast.cgi). Closely related sequenced organisms were found to be *Staphylococcus saprophyticus* subsp. *saprophyticus* ATCC 15305 (genome: 342451.11) with a score of 518, and *Staphylococcus equorum* subsp. *equorum* Mu2 with a score of 512 (genome: 1159488.5). Identification of the urease (EC 3.5.1.5) encoding operon (*ureABCDEFG*) supports the experimental evidence of ureolytic activity. The operon included genes encoding the alpha subunit (*ureC*), beta (*ureB*), and gamma subunits (*ureA*), as well as accessory proteins (*ureDEFG*). Additionally, genes encoding an alternate pathway for urea degradation and ammonia generation, involving allophanate hydrolase (EC 3.5.1.54) and urea carboxylase (EC 6.3.4.6) were found. Antimicrobial resistance genes were identified and similar to those found in other *Staphylococcus* spp. such as *S. aureus* (7). Particular genes of interest were unique to this isolate compared to *S. aureus* RF122 likely resulting from stressors in the poultry litter environment; genes for arsenic and copper resistance (ARC3 and protein D, respectively), ammonia assimilation (ammonium transporter), phosphorus metabolism (exopolyphosphatase), and stress response genes (detoxification, osmotic, oxidative).

### Accession number(s).

This whole-genome shotgun project has been deposited at DDBJ/EMBL/GenBank under the accession no. LGPC00000000. The version described in this paper is the first version, LGPC01000000.
